# SIRT1 mediates nutritional regulation of SREBP-1c-driven hepatic PNPLA3 transcription via modulation of H3k9 acetylation

**DOI:** 10.1186/s41021-022-00246-1

**Published:** 2022-05-27

**Authors:** Xiao Xu, Xiaojie Deng, Yunzhi Chen, Wen Xu, Fen Xu, Hua Liang

**Affiliations:** 1 Department of Endocrinology and Metabolism, Guangdong Provincial Key Laboratory of Diabetology, The Third Affiliated Hospital, Sun Yat-Sen University, Guangzhou, 510630 People’s Republic of China; 2 Department of Emergency, Zhujiang Hospital, Southern Medical University, 510280 Guangzhou, People’s Republic of China

**Keywords:** SIRT1, PNPLA3, SREBP-1c, H3K9ac, Gene regulation

## Abstract

**Background:**

Patatin-like phospholipase domain containing 3 (PNPLA3) is the main nonalcoholic fatty liver disease (NAFLD) susceptibility. Its expression is regulated tightly by nutritional and energy status, but the mechanism of epigenetic regulation of PNPLA3 gene by nutritional dietary factors has not been reported. Here, we investigated the effect and mechanism of Sirtuin 1 (SIRT1) regulated H3K9 deacetylation on PNPLA3 transcriptional expression in vivo and in vitro.

**Methods:**

Mouse models of fasting/re-feeding transition and nonalcoholic fatty liver induced by high Sucrose diet were constructed; and HepG2 cells were treated with serum- and glucose-free medium or exposed to high glucose and high insulin, to generate fasting and high-glucose-induced lipid deposition cell states. Enrichment levels of histone H3K9 acetylation and sterol responsive element binding protein-1c (SREBP-1c) at the PNPLA3 promoter were observed by ChIP-qPCR. PNPLA3 gene expression was detected by real-time PCR; SIRT1 protein expression was detected by western blot. And lipid deposition was detected by Oil Red O.

**Results:**

H3K9ac levels at SRE regions of PNPLA3 promoter were found to be decreased in mice during fasting and increase during refeeding, and increased in mice with NAFLD induced by high-sucrose diet. The change pattern of PNPLA3 promoter H3K9Ac physiologically (fasting/refeeding) and pathologically was consistent with that of PNPLA3 gene expression, but opposite to that of SIRT1 protein expression. In HepG2 cells, overexpression of SIRT1 inhibited high-glucose induced hyper-acetylation of H3K9 at PNPLA3 promoter, and silent expression of SIRT1 suppressed fasting-induced hypo-acetylation of H3K9. Overexpression of SIRT1 prevented basal and SREBP-1c-driven PNPLA3 gene expression and also prevented the endogenous binding of SREBP-1c to PNPLA3.

**Conclusions:**

We first preliminarily revealed SIRT1 may regulate PNPLA3 gene expression by affecting SREBP-1-driven transcription via acetylation modification of H3K9.

**Supplementary Information:**

The online version contains supplementary material available at 10.1186/s41021-022-00246-1.

## Introduction

Multiple lines of evidence suggest that parathin-like phospholipase structural domain 3 (PNPLA3), a member of the parathin-like phospholipase family, is expressed primarily in the liver and adipose tissue and is a key factor in the pathogenesis of nonalcoholic fatty liver disease (NAFLD) [1, 2]. Genome wide association studies (GWAS) and later other studies have reported that PNPLA3 gene polymorphism I148M (rs738409, C > G) is strongly associated with the full disease spectrum of NAFLD, including simple steatosis, steatohepatitis, cirrhosis, and hepatocellular carcinoma [3, 4].

PNPLA3 shows a biphasic effect of lipid synthesis and lipolysis in vitro [5] PNPLA3 expression is nutritionally regulated, decreasing during fasting and increasing after fasting and re-feeding [6]. PNPLA3 is highly expressed in the liver of patients with NAFLD [7] and mouse models of NAFLD induced by high-fat or high-carbohydrate diets or genetic defects [8]. The mechanisms of PNPLA3 gene regulation are not fully understood, but sterol responsive element binding protein-1c (SREBP-1c) is known to be a direct transcription factor of the PNPLA3 gene [6, 9–11]. Transcriptional and epigenetic mechanisms are known to jointly regulate gene expression. However, the epigenetic mechanisms of the PNPLA3 gene regulation are unknown.

The University of California-Santa Cruz (UCSC) Genome Browser Database shows that the promoters of the human and rodent PNPLA3 genes are DNAase hypersensitive sites and have a typical chromatin structure and abundant H3K9ac, H3K4me3, and H3K27ac modification sites [12]. Previous studies have reported that PNPLA3 expression in mice with acute or chronic alcoholic liver injury was associated with histone H3K9ac levels. This suggests that PNPLA3 gene expression may be regulated by the epigenetic mechanism of histone acetylation [13]. Sirtuin 1 (SIRT1) is a deacetylase that inhibits gene expression by promoting promoter-associated histone deacetylation to attenuate promoter binding to transcription factors [14] and plays an important role in the development of NAFLD. The expression of SIRT1 is in contrast to the expression pattern of PNPLA3 on physiology (fasting/re-feeding) and pathology (NAFLD) [15, 16]. Whether SIRT1 is involved in the mechanism of epigenetic regulation of PNPLA3 gene by nutritional dietary factors has not been reported. Therefore, the study aimed to investigate the effect of SIRT1-induced H3K9 deacetylation on hepatic PNPLA3 transcriptional expression and its related mechanism.

## Materials and methods

### Animal model and experimental protocols

All mice were maintained at 22 ± 2 °C and 50 ± 5% relative humidity with a 12:12 h light: dark cycle and had ad libitum access to water. The animal protocol was approved by the Sun Yat-Sen University, Institutional Animal Care and Use Committee.

In energy transition experiments, 7–8-week-old C57BL/6 mice were randomly divided into an ad libitum diet i.e. control group (Ctrl, *n* = 9), fasted group (Fasted, *n* = 9) and refed group (Refed, *n* = 6). The control mice were given ad libitum diet (AIN93M; Nutritional Animal Feed High-Tech Co., Ltd., Nantong, China), the fasted mice were given fasting for 24 h, and the refed mice were given refeeding with a high sugar diet (AIN93M, sucrose content modified to 65%) for 12 h after 24 h of fasting.

For establishment of NAFLD model, C57BL/6 mice (*n* = 10) were randomly divided into a normal chow group (Ctrl, *n* = 5) and a high sucrose diet group (HSD, *n* = 5), and fed for a total of 10 weeks.

### Oil red O staining

Liver tissue was fixed in 10% formalin solution, embedded in embedding medium, and sectioned onto slides with a frozen sectioning machine. The slides were then fixed in 70% ethanol and placed in diluted Oil Red O dye. The specimens were immersed in 60% ethanol. Hematoxylin was then added to stain the cell nuclei. The slides were mounted and observed at 200 × magnification using a Leica DMIL LED inverted microscope (Leica, Germany).

### Cell culture and treatment

HepG2 cells were cultured in Dulbecco's modified Eagle medium (DMEM) containing 5.5 mM glucose, 10% (vol/vol) fetal bovine serum (FBS), 100 units/mL penicillin, and 100 mg/mL streptomycin at 37 °C in a humidified atmosphere containing 5% CO_2_. A cellular model to simulate fasting/refeeding in mice were established by culturing HepG2 cells in DMEM containing 5.5 mM glucose and 10% FBS for 24 h firstly, and then switched to glucose- and serum- free medium for 12 h, and followed by additional 12-h incubation with re-addition of 25 mM glucose. To mimic HSD-induced lipid deposition status, cells were cultured in DMEM containing 25 mM glucose and 100 nM insulin for 24 h.

### Plasmid construction and transfection

Expression plasmids including pcDNA3.1-SIRT1, pcDNA3.1-SREBP-1c, and siRNA oligoribonucleotides target SIRT1 (si-SIRT1, 5-GGAAAUAUAUCCUGGACAATT-3) were generated. Plasmids were transfected using FuGene HD (Roche, USA) and siRNAs were transfected by the X-treme GENE siRNA transfection reagent (Roche, USA). The efficiency of siRNA knockdown was shown in Fig. S1.

### Chromatin immunoprecipitation (ChIP) assay

Chromatin was prepared from liver tissue or HepG2 cells. Chromatin was ultrasonically sheared using a Bioruptor UCD-200 (Diagenode, Belgium), and then immunoprecipitated with anti-H3k9Ac (9649 s, CST), anti-SREBP-1c (sc-13551, Santa Cruz). Normal rabbit IgG (B900610, Proteintech) was used as a mock antibody for negative control. After immunoprecipitating, reverse transcription quantitative polymerase chain reaction (qPCR) was performed. The primers were designed to amplify the reported SREBP-1c binding region (SRE) in mouse and human PNPLA3 promoter [10, 11]. The primer sequences are as follow: mouse: forward 5’-CCTCCCACTGGCTTATTTGC-3’, reverse: 5’-CCGGTGTTGCTGCTCTGAG-3’, human: forward 5’- GCCATCGCCCTCCC AG-3’, reverse: 5’-GGGTGGGGACGACGT-3’. The recruitment is expressed as fold enrichment over IgG (mock).

### Real-time quantitative polymerase chain reaction (RT-qPCR)

Extraction of total RNA was performed using the RNeasy Plus Mini Kit (74,134, Qiagen, Hilden, Germany). The RNA concentration and purity were determined using a Nanodrop2000 spectrophotometer (Thermo Fisher, Waltham, MA, USA), RNA was reverse-transcribed into cDNA using the 5 × All-In-One RT Master Mix Kit (G490; ABM, Richmond, BC, Canada). The real-time fluorescent qPCR was performed using a LightCycler480 SYBR Green I Master kit (Cat.No.04707516001, Roche Diagnostics, Mannheim, Germany) with the LightCycler480II Real-Time PCR system. β-actin was used as internal reference. The primer sequence of mouse PNPLA3 and β-actin genes are as follow, PNPLA3: forward 5’-ACGTGCTGGTGTCTGAGTTCC-3’, reverse: 5’-AGGGACGTT GTCGCTCACTC-3’; β-actin: forward 5’-TGCTATGTTGCCCTAGACTTCG-3’; reverse 5’-GTTGGCATAGAGGTCTTTACGG -3’. The primer sequence of human PNPLA3 and β-actin gens are as follow, PNPLA3: forward 5’- GGCATCTCTCTTACCAGAGTGT C-3’, reverse: 5’- GGCATCCACGACTTCGTCTT -3’; β-actin: forward 5’- GGCTGTATTCCCCTCCATCG -3’; reverse5’-GTTGGCATAGAGGTCTTTACGG -3’.

### Western blot analysis

Total protein was extracted from liver samples or HepG2 cells using T-PER™ tissue protein extraction reagent (Pierce cat. 78,510). Protein was divided into SDS-PAGE (5% concentrated gel and 10% separated gel) and transferred to PVDF membrane. After sealing with 5% milk diluent, incubating with primary antibodies for SIRT1 (#2028, CST) and fluorescent secondary antibodies, the membrane was visualized using the Odyssey infrared fluorescence imaging system (Licor, USA).

### Statistical analysis

Statistical analysis was performed using the Statview 5.0. Square root transformation was used to normalize the skewed data. Data are shown as the means ± standard deviation (SD) values of three independent duplicate experiments. Student t- test was used to analyze the statistical difference for comparisons between two groups. One-way ANOVA followed by Fisher’s least significant difference (LSD) test was used to determine statistical significance for comparisons among multiple groups.

## Results

### Liver SIRT1 expression had the opposite trend with PNPLA3 gene expression and PNPLA3 promoter histone H3K9 acetylation in fasting/refeeding mice and high-fructose-induced NAFLD mice.

**Fig. 1:**
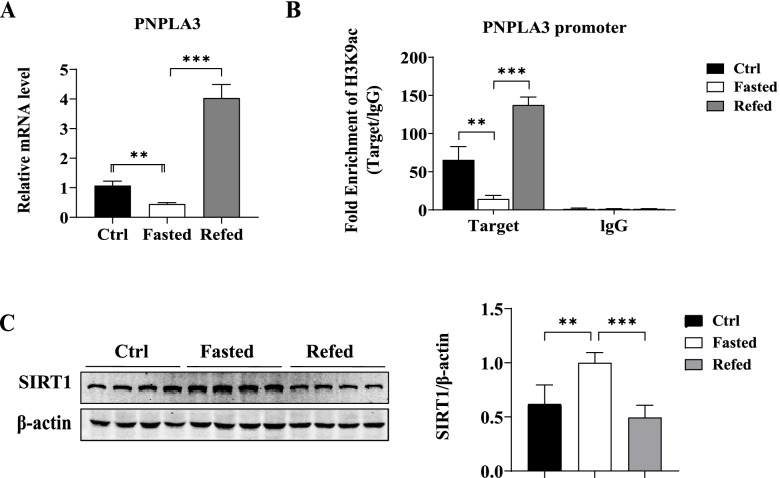
Hepatic PNPLA3 gene and SIRT1 protein expression and H3K9ac level of PNPLA3 promoter in C57BL/6 mice during the fasting/re-feeding transition. **A** Relative mRNA level of liver PNPLA3 measured by qPCR (*N* = 6 mice per group). **B** The recruitment of H3K9ac onto the PNPLA3 promoter detected by ChIP-qPCR using an anti-H3k9Ac antibody. Rabbit IgG was used as a mock antibody for negative control. The recruitment is expressed as fold enrichment over IgG (*N* = 4 mice per group). **C** The protein level of liver SIRT1 detected by western blot. Quantitative analysis of gray value on bands was performed using Image-Pro Plus software. Quantitative data are presented as the means ± SD (*N* = 3 independent experiments). Ctrl: mice fed a normal chow diet; Fasted: mice fasted for 24 h; Refed: mice refed a high sucrose diet for 12 h after 24 h of fasting. **P < 0.01, ***P < 0.001

**Fig. 2:**
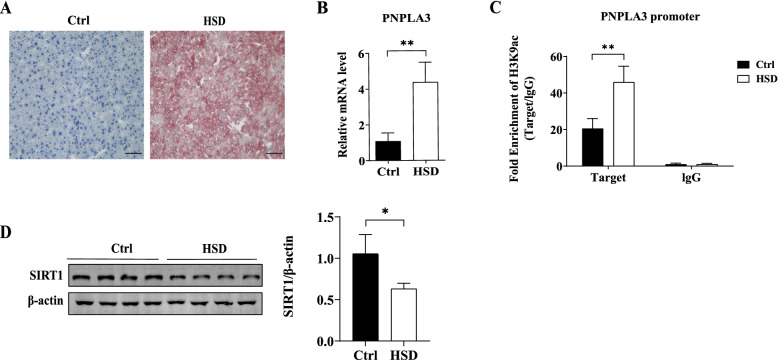
Hepatic PNPLA3 gene and SIRT1 protein expression and H3K9ac level of PNPLA3 promoter in C57BL/6 mice with NAFLD induced by high-sucrose diet. **A** Visualized oil red O staining using a research-grade inverted microscope at 200 × magnification. **B** Relative mRNA level of liver PNPLA3 measured by qPCR (*N* = 5 mice per group)..**C** The recruitment of H3K9ac onto the PNPLA3 promoter detected by ChIP-qPCR using an anti-H3k9Ac antibody. Rabbit IgG was used as a mock antibody for negative control. The recruitment is expressed as fold enrichment over IgG (*N* = 4 mice per group). **D** The protein level of liver SIRT1 detected by western blot. Quantitative analysis of gray value on bands was performed using Image-Pro Plus software. Quantitative data are presented as the means ± SD (*N* = 3 independent experiments). Ctrl: control mice fed a normal chow diet, HSD: mice fed a high sucrose diet. *P < 0.05, **P < 0.01

PNPLA3 gene expression, H3K9 acetylation (H3K9ac) level of the PNPLA3 promoter, and SIRT1 protein expression were detected in mice during fasting and refeeding transition (Fig. 1), as well as in HSD-fed mice (Fig. 2). The expression of PNPLA3 mRNA and the level of H3K9ac on the PNPLA3 promoter region were significantly decreased during fasting and increased during refeeding after a fast compared with control mice fed ad libitum with normal chow diet (Fig. 1A and Fig. 1B). SIRT1 protein expression showed opposite pattern to that of levels of PNPLA3 mRNA and H3K9ac, i.e., SIRT1 expression increased during fasting and decreased during refeeding (Fig. 1C).

In HSD-fed mice, markedly lipid accumulation in liver was observed by oil red staining (Fig. 2A). PNPLA3 mRNA levels were upregulated by 4.05 folds in HSD mice compared to control mice (*P* = 0.0012 vs. Ctrl, Fig. 2B). HSD also induced significant increase in H3K9ac enrichment at the PNPLA3 promoter (2.24-fold increase, *P* = 0.0041 vs. Ctrl, Fig. 2C) and downregulation of SIRT1 protein expression (Fig. 2D).

### SIRT1 regulated histone H3K9 acetylation level at the PNPLA3 gene promoter

**Fig. 3:**
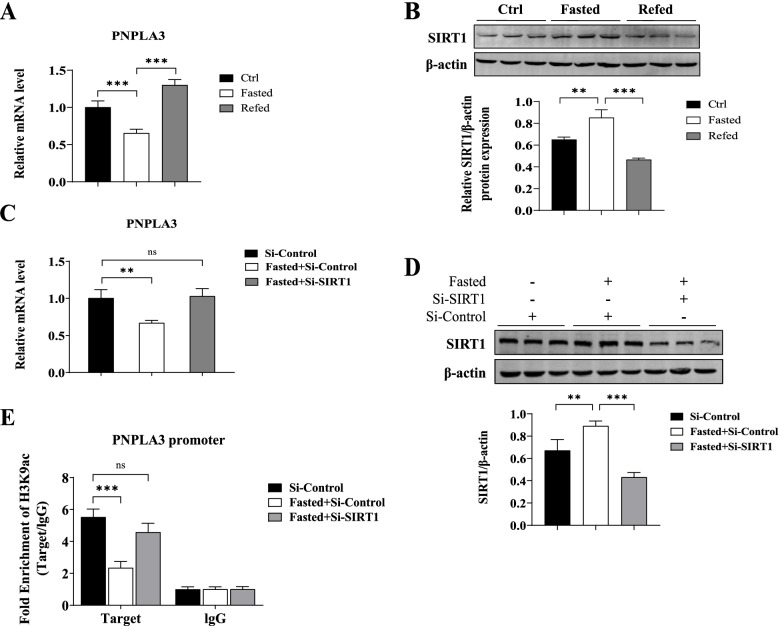
Gene expression of PNPLA and H3K9ac level of PNPLA3 promoter were regulated by SIRT1 in starved (fasted) HepG2 cells. **A** and **B** Relative mRNA of PNPLA3 (**A**) measured by qPCR and protein level of SIRT1 (**B**) detected by western blot in a cellular model to simulate fasting/refeeding in mice. **C-E** HepG2 cells were pretreated with siRNA-Control or si-SIRT1, followed by treatment of fasting and refeeding. Relative mRNA of PNPLA3 (**C**) was measured by qPCR. The protein level of SIRT1 (**D**) was detected by western blot. The recruitment of H3K9ac onto the PNPLA3 promoter (**E**) was detected by ChIP-qPCR using an anti-H3k9Ac antibody. Rabbit IgG was used as a mock antibody for negative control. The recruitment is expressed as fold enrichment over IgG. Biological replicates (*N* = 3) were performed per group. Quantitative data are presented as the means ± SD (*N* = 3 independent experiments). Quantitative analysis of gray value on bands of western blot was performed using Image-Pro Plus software. Ctrl: cells incubated in media containing 5.5 mM glucose and 10% fetal bovine serum; Fasted: cells incubated in glucose- and serum-free medium for 12 h; Refed: cells incubated in glucose- and serum- free medium for 12 h and followed by additional 12-h incubation with re-addition of 25 mM glucose. **P < 0.01; ***P < 0.001

A HepG2 cellular model to simulate fasting/refeeding in mice were established. As in Fig. 3A, PNPLA3 mRNA expression was significantly reduced upon cell starvation (fasting) induced by incubating in serum- and glucose-free medium for 12 h, and significantly increased upon serum and 25 mM glucose re-addition, exhibiting a similar pattern of expression to that of the liver of fasted/re-fed mice. Similarly, the SIRT1 protein expression characteristics of starvation and reinduction of serum and glucose were also identical to those of mice during fasting/re-feeding (Fig. 3B). We next performed experiments to elucidate the role of SIRT1 in the regulation of H3K9ac levels at the PNPLA3 promoter. HepG2 cells pretreated with siRNA-Control or si-SIRT1 were incubated in media containing 5.5 mM glucose and 10% fetal bovine serum for 24 h, followed by an additional 12-h glucose- and serum-free incubation to induce cell starvation. As shown in Fig. 3C and 3D, cell starvation resulted in a 33.2% downregulation of PNPLA3 gene expression (*P* = 0.0027 vs. Si-Control, Fig. 3C) and a significant upregulation of SIRT1 protein expression (Fig. 3D). The level of H3K9ac of the PNPLA3 promoter was significantly reduced upon cell starvation (48% reduction, P = 0.0002 vs. Si-Control, Fig. 3E), being consistent with changes in PNPLA3 gene expression. Silence of SIRT1 expression with siRNA (Fig. 3D) prevented the downregulation of PNPLA3 gene expression (Fig. 3C) and the decrease of H3K9ac level (Fig. 3E) induced by cell starvation.

**Fig. 4:**
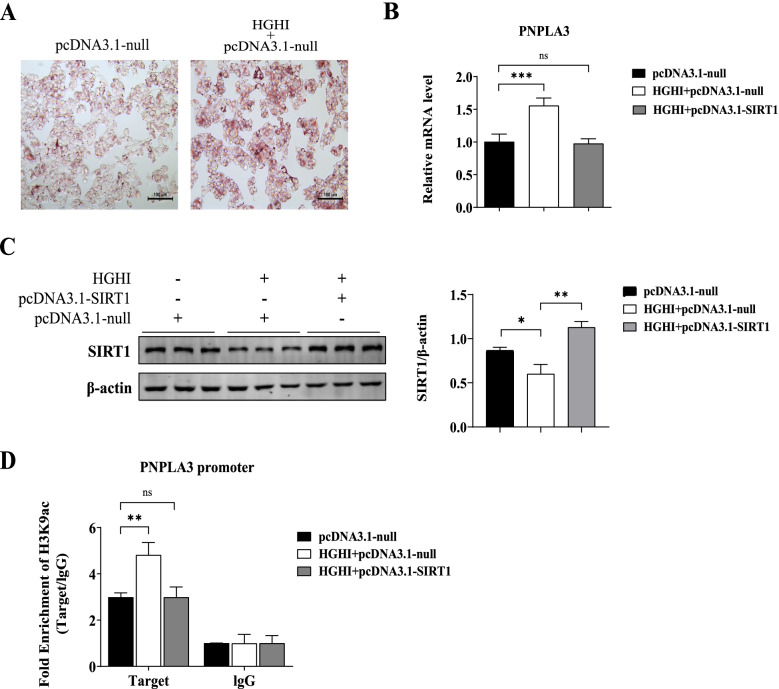
Gene expression of PNPLA and H3K9ac level of PNPLA3 promoter were regulated by SIRT1 in HGHI-treated HepG2 cells. HepG2 cells pretreated with pcDNA3.1-null or pcDNA3.1-SIRT1 were incubated in media containing 25 mM glucose. **A** Visualized oil red O staining using a research-grade inverted microscope at 200 × magnification. **B** Relative mRNA of PNPLA3 measured by qPCR. **C** The protein level of SIRT1 detected by western blot. Quantitative analysis of gray value on bands was performed using Image-Pro Plus software. **D** The recruitment of H3K9ac onto the PNPLA3 promoter detected by ChIP-qPCR using an anti-H3k9Ac antibody. Rabbit IgG was used as a mock antibody for negative control. The recruitment is expressed as fold enrichment over IgG. Biological replicates *(N* = 3) were performed per group. Quantitative data are presented as the means ± SD (*N* = 3 independent experiments).*P < 0.05; **P < 0.01; ***P < 0.001

On the other hand, HepG2 cells exposed to 25 mM glucose and 100 nM insulin (high glucose and high insulin, HGHI), compared with cells without HGHI treatment, exhibited the markedly higher lipid droplet area shown by oil red staining (Fig. 4A), an upregulation of PNPLA3 gene expression (1.55-fold upregulation, *P* = 0.0006 vs. pcDNA3.1-null, Fig. 4B), and a downregulation SIRT1 protein expression (Fig. 4C). The HGHI-induced upregulated expression of PNPLA3 gene was prevented (Fig. 4B) by overexpression SIRT1 via transfection of pcDNA3.1-SIRT1 (Fig. 4C). Increased enrichment of H3K9ac at the PNPLA3 promoter was observed in cells treated with HGHI (1.51-fold increase, *P* = 0.0414 vs. pcDNA3.1-null, Fig. 4D). And overexpression SIRT1 can antagonized the HGHI-induced increase in H3K9ac level at PNPLA3 promoter (Fig. 4D).

### SIRT1 overexpression inhibited PNPLA3 gene expression via decreasing SREBP-1c-PNPLA3 binding

**Fig. 5:**
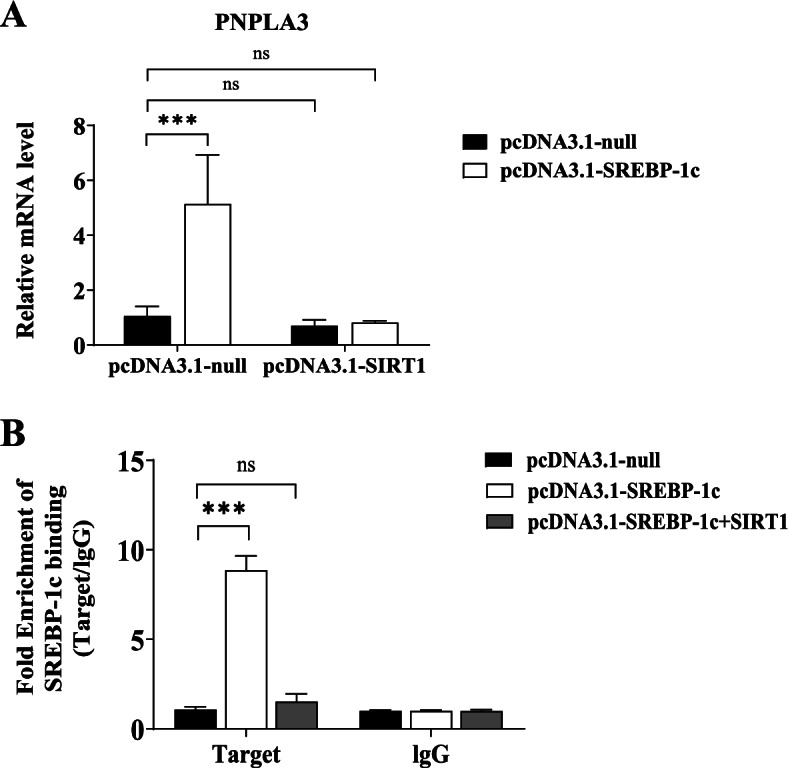
SIRT1 regulates SREBP-1c-driven PNPLA3 gene expression in HepG2 cells. **A** Effect of SIRT1 on basal and SREBP-1c-driven PNPLA3 gene expression. HepG2 cells were transfected respectively or cotransfected with pcDNA3.1- SREBP-1c and SIRT1. PNPLA3 mRNA levels were measured by qPCR. **B** Effect of SIRT1 on SREBP-1c enrichment on the PNPLA3 promoter. HepG2 cells were transfected with pcDNA3.1-SREBP and pcDNA3.1-SIRT1 alone, or were cotransfected both together for 24 h. The endogenous binding of SREBP-1c and PNPLA3 promoter was detected by ChIP-qPCR, which was performed using an anti-SREBP-1c antibody. Rabbit IgG was used as a mock antibody for negative control. The recruitment is expressed as fold enrichment over IgG. Biological replicates (*N* = 3) were performed per group. Quantitative data are presented as the means ± SD (*N* = 3 independent experiments). ***P < 0.001

To understand the effect of SIRT1 on basal and SREBP-1c-driven PNPLA3 gene expression, we examined PNPLA3 gene expression in HepG2 cells overexpressing SIRT1 or SREBP-1c alone and co-expressing SIRT1 and SERBP-1c. PNPLA3 gene expression was increased by 4.86-fold after overexpression of SREBP-1c (*P* < 0.0001 vs. pcDNA3.1-null, Fig. 5A). Overexpression of SIRT1 significantly suppressed the increase in PNPLA3 gene expression induced by overexpression of SREBP-1c, although overexpression of SIRT1 alone had no significant effect on PNPLA3 expression (Fig. 5A). The enrichment of SREBP-1c on the PNPLA3 promoter SRE region was further examined to elucidate whether SIRT1 regulates the binding of SREBP-1c to the PNPLA3 gene. As shown in Fig. 5B, in HepG2 cells, overexpression of SREBP-1c induced an 8.2-fold increase in SREBP-1c enrichment on the PNPLA3 promoter (*P* < 0.0001 vs. pcDNA3.1-null). Upon co-transfection of SREBP-1c and SIRT1, SREBP-1c overexpression-induced SREBP-1c enrichment on the PNPLA3 promoter was markedly suppressed.

## Discussion

In the present study, we report the epigenetic mechanism by which energy changes regulate hepatic PNPLA3 gene expression, namely by regulating PNPLA3 promoter H3K9Ac levels through SIRT1 to affect SREBP-1c-driven PNPLA3 gene expression.

PNPLA3 is the main NAFLD-susceptibility gene. Its polymorphism I148M is closely associated with the onset and progression of NAFLD by probably reducing lipid hydrolysis or increasing lipid synthesis and promoting endoplasmic reticulum stress-related inflammation [17]. PNPLA3 gene expression is tightly regulated by nutritional status, but the exact underlying molecular mechanisms are not clear yet. In the present study, we report that SIRT1 is involved in the regulation of PNPLA3 gene expression in physiological and pathological states, and the mechanism may involve SIRT1 regulation of PNPLA3 promoter H3K9Ac levels affecting its binding to SREBP-1c.

According to UCSC database, H3K9ac modifications are highly enriched in the human and rodent PNPLA3 promoters, so we investigated the response of PNPLA3 gene H3K9ac levels to physiological and pathological energetic changes. We found that in C57BL/6 J mice, H3K9ac levels of the hepatic PNPLA3 gene were tightly regulated by energy changes, i.e., H3K9ac levels were suppressed during fasting and elevated after refeeding and long-term HSD feeding, suggesting that histone acetylation is involved in the mechanism of energy regulation of PNPLA3 gene expression. Histone H3K9 is a major target of SIRT1, which belongs to the NAD + -dependent class III deacetylase sirtuin family and plays an important role in the regulation of lipid and glucose metabolism associated with nutritional and hormonal signaling [18] and therefore influences the development and progression of various metabolic disorders such as NAFLD and obesity [19]. We showed that in C57BL/6 J mice, SIRT1 expression is upregulated during fasting and downregulated after refeeding and long-term HSD feeding in a pattern opposite to PNPLA3 gene expression and its promoter H3K9ac levels, suggesting that SIRT1 may be involved in regulation of PNPLA3 gene expression by affecting H3K9ac levels.

We and others have shown that SREBP-1c is a direct transcription factor regulating the PNPLA3 gene [6, 9–11]. Insulin-induced upregulation of SREBP-1c is involved in the mechanism of regulation of PNPLA3 expression by nutritional status [6, 11]. In general, acetylation of histones contributes to the dissociation of DNA and histone octamers and the relaxation of nucleosome structure, facilitating the binding of various transcription factors to DNA site-specific interactions to activate gene transcription [19]. Given that we detected H3K9ac enrichment in the SRE region of the PNPLA3 gene, this suggests that acetylation modification of H3K9 may affect SREBP-1c-driven transcription of the PNPLA3 gene. In the present study, SIRT1 overexpression inhibited SREBP-1c overexpression-induced PNPLA3 expression upregulation in vitro by reducing SREBP-1c binding to the PNPLA3 gene promoter, suggesting that SIRT1 is a key regulator of SREBP-1c-driven PNPLA3 transcriptional expression.

Recent studies have shown that intermittent fasting has an ameliorative effect on fatty liver [20, 21]. PNPLA3 and SIRT1 expression are regulated by fasting/refeeding status and are both involved in the pathogenesis of NAFLD. Our previous study found that SIRT1 is a key factor in mediating the weight loss response and reducing hepatic steatosis by GLP-1RA [22, 23]. Therefore, exploring the mechanisms by which SIRT1 regulates PNPLA3 expression in energy metabolism will bring new insights into the treatment of NAFLD.

Finally, the potential mechanisms by which histone acetylation regulates PNPLA3 gene expression are not well studied in the current study. In addition, other deacetylases, such as SIRT6, HDAC2 and HDAC8, also have different degrees of H3K9 deacetylation, so we cannot exclude the regulatory role of other deacetylases on PNPLA3 gene expression.

## Conclusions

In conclusion, SIRT1 regulates the level of H3K9 acetylation at the PNPLA3 promoter and thus affects SREBP-1c-driven PNPLA3 gene expression may be involved in the mechanism of energy regulation of PNPLA3 gene expression.

## Supplementary Information

**Additional file 1.** 

## Data Availability

All the original data of the article and the archived files of the experiment process could be obtained from the corresponding author with permission.

## References

[1] Baulande S, Lasnier F, Lucas M, Pairault J (2001). Adiponutrin, a transmembrane protein corresponding to a novel dietary- and obesity-linked mRNA specifically expressed in the adipose lineage. J Biol Chem.

[2] Bruschi FV, Claudel T, Tardelli M, Caligiuri A, Stulnig TM, Marra F (2017). The PNPLA3 I148M variant modulates the fibrogenic phenotype of human hepatic stellate cells. Hepatology.

[3] Li  YY (2012). Genetic and epigenetic variants influencing the development of nonalcoholic fatty liver disease. World J Gastroenterol.

[4] Eslam M, Valenti L, Romeo S (2018). Genetics and epigenetics of NAFLD and NASH: Clinical impact. J Hepatol.

[5] Bruschi FV, Tardelli M, Claudel T, Trauner M (2017). PNPLA3 expression and its impact on the liver: current perspectives. Hepat Med.

[6] Huang Y, He S, Li JZ, Seo YK, Osborne TF, Cohen JC (2010). A feed-forward loop amplifies nutritional regulation of PNPLA3. Proc Natl Acad Sci USA.

[7] Adams LA, White SW, Marsh JA, Lye SJ, Connor KL, Maganga R (2013). Association between liver-specific gene polymorphisms and their expression levels with nonalcoholic fatty liver disease. Hepatology.

[8] Smagris E, BasuRay S, Li J, Huang Y, Lai KM, Gromada J (2015). Pnpla3I148M knockin mice accumulate PNPLA3 on lipid droplets and develop hepatic steatosis. Hepatology.

[9] Dubuquoy C, Robichon C, Lasnier F, Langlois C, Dugail I, Foufelle F (2011). Distinct regulation of adiponutrin/PNPLA3 gene expression by the transcription factors ChREBP and SREBP1c in mouse and human hepatocytes. J Hepatol.

[10] Qiao A, Liang J, Ke Y, Li C, Cui Y, Shen L (2011). Mouse patatin-like phospholipase domain-containing 3 influences systemic lipid and glucose homeostasis. Hepatology.

[11] Liang H, Xu J, Xu F, Liu H, Yuan D, Yuan S (2015). The SRE Motif in the Human PNPLA3 Promoter (-97 to -88 bp) Mediates Transactivational Effects of SREBP-1c. J Cell Physiol.

[12] Sookoian S, Pirola CJ (2012). PNPLA3, the triacylglycerol synthesis/hydrolysis/storage dilemma, and nonalcoholic fatty liver disease. World J Gastroenterol.

[13] Restrepo RJ, Lim RW, Korthuis RJ, Shukla SD (2017). Binge alcohol alters PNPLA3 levels in liver through epigenetic mechanism involving histone H3 acetylation. Alcohol.

[14] Wang RH, Li C, Deng CX (2010). Liver steatosis and increased ChREBP expression in mice carrying a liver specific SIRT1 null mutation under a normal feeding condition. Int J Biol Sci.

[15] Zhu Y, Yan Y, Gius DR, Vassilopoulos A (2013). Metabolic regulation of Sirtuins upon fasting and the implication for cancer. Curr Opin Oncol.

[16] Chalkiadaki A, Guarente L (2012). Sirtuins mediate mammalian metabolic responses to nutrient availability. Nat Rev Endocrinol.

[17] Yuan S, Liu H, Yuan D, Xu J, Chen Y, Xu X (2020). PNPLA3 I148M mediates the regulatory effect of NF-kB on inflammation in PA-treated HepG2 cells. J Cell Mol Med.

[18] Nassir F, Ibdah JA (2016). Sirtuins and nonalcoholic fatty liver disease. World J Gastroenterol.

[19] Lee J, Kim Y, Friso S, Choi SW (2017). Epigenetics in non-alcoholic fatty liver disease. Mol Aspects Med.

[20] Marinho TS, Ornellas F, Barbosa-da-Silva S, Mandarim-de-Lacerda CA, Aguila MB (2019). Beneficial effects of intermittent fasting on steatosis and inflammation of the liver in mice fed a high-fat or a high-fructose diet. Nutrition.

[21] Hatori M, Vollmers C, Zarrinpar A, DiTacchio L, Bushong EA, Gill S (2012). Time-restricted feeding without reducing caloric intake prevents metabolic diseases in mice fed a high-fat diet. Cell Metab.

[22] Zheng X, Xu F, Liang H, Cao H, Cai M, Xu W (2017). SIRT1/HSF1/HSPs Pathway is Essential for Exenatide-alleviated Lipid-induced Hepatic Endoplasmic Reticulum Stress. Hepatology.

[23] Xu F, Li Z, Zheng X, Liu H, Liang H, Xu H (2014). SIRT1 Mediates the Effect of GLP-1 Receptor Agonist Exenatide on Ameliorating Hepatic Steatosis. Diabetes.

