# Supplementary material for: SIRT1 mediates nutritional regulation of SREBP-1c-driven hepatic PNPLA3 transcription via modulation of H3k9 acetylation
Source: Genes Environ. 2022 May 27;44:18. doi: 10.1186/s41021-022-00246-1 (PMC9137095; doi:10.1186/s41021-022-00246-1)
Supplement: Supplementary file 1 — Additional file 1. [file 41021_2022_246_MOESM1_ESM.pdf]

Figure Legends

Fig. S1 The knockdown efficiency of siRNA targeting SIRT1 in HepG2 cells. **A** qPCR analysis of SIRT1 knockdown efficiency. Data represent the means ± SD of three independent experiments. **B** Western blot analysis of SIRT1 knockdown efficiency in HepG2 cells. Quantitative analysis of gray value on bands was performed using Image-Pro Plus software. *P <0.05, ***P <0.001.

Fig. S1

A

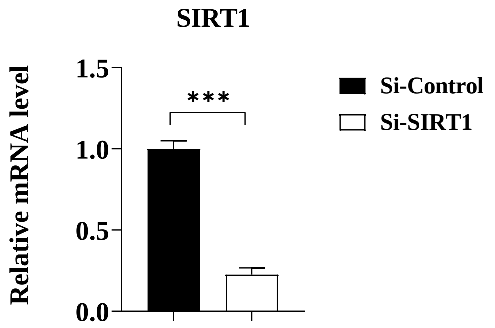
SIRT1
Relative mRNA level
1.5
1.0
0.5
0.0
***
Si-Control
Si-SIRT1

B

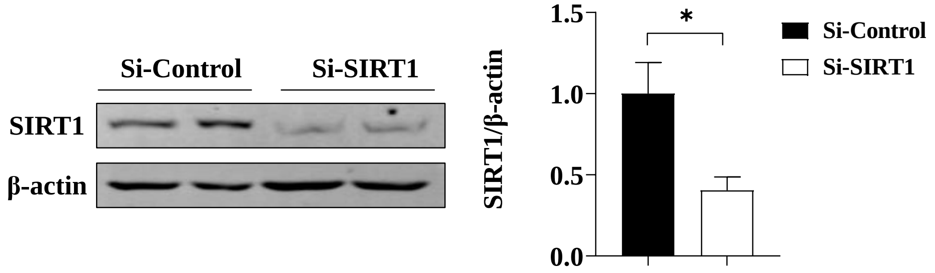
Si-Control
Si-SIRT1
SIRT1
β-actin
SIRT1/β-actin
1.5
1.0
0.5
0.0
*
Si-Control
Si-SIRT1
